# Adolescence as a key developmental window for nutrition promotion and cardiometabolic disease prevention

**DOI:** 10.1038/s44324-025-00082-1

**Published:** 2025-10-10

**Authors:** Bianca Carducci, Zheng Hao Chen, Susan C. Campisi, Kozeta Miliku

**Affiliations:** 1 Columbia Climate School, Columbia University, New York, NY USA; 2 Department of Nutritional Sciences, Temerty Faculty of Medicine, University of Toronto, Toronto, ON Canada; 3 Neurosciences and Mental Health, The Hospital for Sick Children, Toronto, ON Canada; 4 Nutrition and Dietetics Program, Clinical Public Health Division, Dalla Lana School of Public Health, University of Toronto, Toronto, ON Canada; 5 Department of Medicine, Faculty of Health Sciences, McMaster University, Hamilton, ON Canada

**Keywords:** Diabetes, Metabolic syndrome, Obesity, Metabolism

## Abstract

Adolescence is a key developmental window of opportunity for nutrition promotion and cardiometabolic disease (CMD) prevention that can reap long-term significant health, economic and social advantages, however it is currently not a focus in the Developmental Origins of Health and Disease (DOHaD) framework. In this perspective, we argue that adolescence should be included in the DOHaD framework, by examining current evidence on the relationship between adolescent nutrition and risk factors for CMDs, physiological mechanisms, and potential interventions.

## Introduction

Adolescence, or the window between 10 and 19.9 years of age, is a critical period of rapid development where adverse exposures (e.g., poor nutrition) can potentially increase the risk of cardiometabolic diseases (CMDs) like obesity, cardiovascular disease, diabetes, and metabolic syndrome later in life^1^. This aligns with The Developmental Origins of Health and Disease (DOHaD) framework, also known as the “Barker hypothesis“^1^, where biological programming during critical periods of development can alter homeostatic mechanisms regulating appetite and energy output. These changes may contribute to long-term metabolic disruptions and, ultimately, lead to CMDs^2^. Traditionally, DOHaD has emphasized the “first 1000 days”—the period from conception to age two years—as a window for nutritional programming and chronic disease prevention. More recently, DOHaD scholars have broadened this view to include adolescence as a biologically sensitive and socially meaningful life stage. For example, Hanson and Gluckman conceptualized adolescence as a key opportunity to influence both individual and intergenerational health trajectories^3^. Paton et al. further detailed how pubertal and post-pubertal transitions represent windows for environmental imprinting^4^.

However, despite these important theoretical contributions, adolescence remains underrepresented in the nutrition-specific operationalization of the DOHaD framework. Compared to early life stages, there are few global nutrition strategies, longitudinal cohorts, or interventional studies that explicitly position adolescence as a target for DOHaD-informed action. This gap is particularly striking given that adolescence is a period marked by increased nutritional demands, hormonal shifts, and behavioral transitions, all of which can influence long-term cardiometabolic disease (CMD) risk^5^. Described as an “invisible epidemic”, the incidence of CMDs and the prevalence of related risk factors such as suboptimal diets are rapidly rising, creating global social and economic burdens^6^. Addressing CMDs has emerged as a global priority in the Sustainable Development Goals (Target 3.4) and was the focus of a third UN high-level meeting^7^; however adolescence has not been considered as a critical window for prioritized interventions.

Adequate nutrition and energy consumption during early adolescence, particularly during puberty, is required for development including muscle mass increase, fat accumulation, brain and linear growth and reproductive maturation^5^. According to the Institute of Medicine Dietary Reference Intakes, macro- and micro-nutrient intakes increase substantially in adolescence, with notable sex-driven differences. Current research on whether adolescents are meeting their nutritional requirements through dietary intake, micronutrient status, or anthropometric measures remains limited. Of the available data, the estimated prevalence of deficiency in at least one of three core micronutrients (iron, zinc and vitamin A) was 69% (95% Uncertainty Interval: 59–78) in non-pregnant women of reproductive age (15–49 years)^8^. Importantly, iron deficiency and iron deficiency anemia account for the majority of Disability Adjusted Life Years (DALYs) (primarily in adolescent females) associated with micronutrient deficiencies in adolescents globally (>2500 DALYs per 100,000 adolescents as of 2015)^9,10^. Adolescents in South Asia and sub-Saharan Africa also appear vulnerable to iodine deficiency, and to a lesser extent, vitamin A deficiency^10,11^.

In addition to micronutrient status, there remains various data gaps on adolescent global dietary intake^12^. The Global School-based Student Health Survey (GSHS) is a nationally representative self-administered questionnaire aimed at understanding behavioral behavioral and lifestyle risk factors of school-going adolescent males and females (primarily 12–17 years), in both high-income and low- and middle-income countries (LMICs). A meta-analysis by Beal et al. found significant heterogeneity between regions administering the GSHS (94 countries) for the mean frequency of consumption of fruit and carbonated soft drinks, but not for vegetable and fast-food consumption^13^. Further, the authors found that 34.5% of adolescents (95% Confidence Interval (CI): 29.4–39.7) consumed fruit less than once per day, 20.6% (95% CI: 15.8–25.9) consumed vegetables less than once per day, 42.8% (95% CI: 35.2–50.7) drank carbonated soft drinks at least once per day, and 46.1% (95% CI: 38.6–53.7) consumed fast food at least once per week^13^. Other attempts to synthesize empirical evidence on nationally representative dietary patterns, ultra-processed foods including sugar and salt consumption, in this population, have been limited to high-income countries (Health Behavior in School-aged Children Survey)^14–16^.

Studies examining the association between adolescent diets and long-term health outcomes suggest that poor quality diets (in terms of adequacy and diversity) and unbalanced dietary patterns in adolescence increase the risk of clinical markers of metabolic syndrome including insulin resistance, dyslipidemia, and obesity, as well as inflammation^17–20^. However, there have been few attempts to synthesize empirical evidence on adolescent nutrition plasticity, especially underlying mechanisms and longitudinal impacts. There is now well-established global evidence on the suite of effective and cost-effective policies, programs and interventions that should be implemented at scale to rapidly curb the burden of CMDs globally^21^. Evidence suggests that several CMD risk factors during adulthood could be prevented with appropriate multisectoral interventions across the life course^22^. Global efforts to examine evidence-informed interventions in children and adolescents focus on long-term, sustainable behavior change enabled by health-promoting environments and supportive policies to prevent the development of unhealthy risk factors and subsequent disease onset in adolescence, adulthood or future generations^22,23^.

This perspective does not aim to redefine the DOHaD model, but to provide evidence and argue for the formal integration of adolescence as a distinct and actionable phase within it, in terms of influencing individual health trajectories. While there have been some studies examining adolescent nutrition as a critical exposure for intergenerational health outcomes, there is limited and inconsistent evidence in our understanding of optimizing adolescent dietary habits for immediate and long-term health outcomes^5^. Therefore, this perspective examines current evidence on the relationship between adolescent nutrition and CMDs, identifies potential mechanisms, recommends evidence-based interventions for early intervention and prevention of CMDs and future research directions for this space.

## Current evidence on the association between adolescent diets and cardiometabolic diseases

Longitudinal evidence, both prospective and retrospective, that observes dietary exposures in normal-weight adolescents on the development or attenuation of risk factors for CMDs such as abdominal obesity, insulin resistance, dyslipidemia and hypertension has suggested that consuming nutrient-dense foods in adolescence, measured through dietary intake or dietary pattern scores is positively associated with lower chronic disease risk in adulthood^24^. Conversely, other studies have found that nutrient-poor diets and consumption of ultra-processed foods are associated with greater body mass index, an increased risk of developing type 2 diabetes markers and inflammation in adulthood^25^. However, most of these studies were conducted in high-income populations in Europe, Australia or United States. For example, findings from the United Kingdom (UK) Avon Longitudinal Study of Parents and Children (ALSPAC) Study, one of the largest European studies, suggest a significant association between a higher dietary inflammatory score during adolescence and higher body mass index (BMI) in adulthood (*N* = 1937), independent of potential confounders such as child sex, family socioeconomic status, and physical activity^26^. Also in the UK, the Determinants of Adolescent Social wellbeing and Health (DASH) Study found that, after adjusting for confounders (e.g., child sex and ethnicity, parental education, physical activity), low fruit intake was associated with a larger waist-to-height ratio (*N* = 665)^27^. Similar to the UK findings, in Australia, the Raine Study (*N* = 667), found that higher intakes of sugar-sweetened beverages (SSB) and diet drinks were associated with higher BMI and waist circumference, and greater odds of living with overweight or obesity, after adjusting for similar models as previous studies in addition to baseline anthropometrics^28^. However, there are also some conflicting results which may be due to different settings (e.g., different European cohorts), methodologies used (e.g., different a-priori approaches to assess dietary intake, different assessment of obesity/adiposity), and model adjustment (e.g., baseline BMI, physical activity). For example, in both the Dortmund Nutritional and Anthropometric Longitudinally Designed (DONALD) Study from Germany (*N* = 226) and the Epidemiological Health Investigation of Teenagers (EPITeen) Study from Portugal (*N* = 1034), no significant associations were observed between glycemic load with BMI^29,30^.

The sex-driven associations between adolescent diet and obesity-related outcomes are also worth noting. For example, in the DONALD Study, it was found that higher animal protein intake during adolescence was associated with higher fat-free mass in adulthood, particularly among females, independent of confounders including baseline fat-free mass index, socioeconomic status, nutrient intakes, but did not adjust for physical activity^31^. Whereas, in the Amsterdam Growth and Health Longitudinal Study (AGAHLS) from the Netherlands (*N* = 238), after adjusting for confounders (e.g., baseline BMI, physical activity, energy intake), a higher intake of SSB during adolescence was associated with higher percent total fat and percent trunk fat among adult males^32^. There are some inconsistent sex-driven associations between vegetable intake and obesity. For example, findings from the Project EAT (Eating and Activity in Teens and Young Adults) from the United States (*N* = 1643) showed a significant association between higher vegetable intake and lower odds of overweight among males, adjusted for socioeconomic status and ethnicity^33^. Meanwhile in the AGAHLS (*N* = 168), lower vegetable intake was associated with higher BMI and skinfold among females, adjusted for physical activity, tobacco use, and fiber intake^34^. Given that sex-driven associations are present in existing evidence, future studies should examine deeper into the moderating role of sex to improve our understanding and explore adjustment models that include physical activity, socioeconomic status and baseline body composition for comparability.

Similar to BMI, high blood pressure and subsequent hypertension, can also track throughout life, leading to a multitude of CMDs^35^. For example, the AGAHLS (*N* = 373) study found a high score of the alternate Mediterranean diet at 13 years of age was significantly associated with both lower systolic and diastolic blood pressure, and lower risk of stiffer carotid arteries after 24 years of follow-up^36^. Micronutrients during adolescence, (e.g. calcium intake in the China Health and Nutrition Survey (CHNS) Study (*N* = 1611)), have been associated with systolic blood pressure and risk of hypertension in adulthood^37^. Both these studies adjusted for physical activity and smoking, while the CHNS study included further covariates in their models (e.g., education, alcohol intake, urbanization, and obesity status). Other studies, such as the EPITeen Study (*N* = 862), did not find any significant associations between *a-posteriori* data-driven dietary patterns and blood pressure measurements, after adjustment for child sex and education^38^. Although there is some supporting evidence on the role of diet during adolescence and adulthood hypertension development, including preclinical outcomes like blood pressure and carotid artery stiffness, further studies are needed.

Sex-specific associations have been reported between adolescent diet and blood pressure. For example, in the DONALD Study (*N* = 206), lower fruit and vegetable intake was associated with higher systolic blood pressure among female adults, while the association between higher salt intake and higher systolic blood pressure was observed among males^39^. Further sex-stratified analyses are needed to fully understand the epidemiology of sex-driven blood pressure development from diet in adolescence.

Findings from prospective cohort studies on adolescence diet and diabetes are limited and inconsistent. In the DONALD Study (*N* = 226), higher intake of high glycemic index during adolescence was associated with higher homeostasis model assessment insulin resistance (HOMA-IR) (adjusted for child sex, baseline BMI, socioeconomic status etc.)^40^. However, the EPITeen Study (*N* = 1034) did not find significant associations with the glycemic load and HOMA-IR, despite similar follow-up times of around 10 years (adjusted for child sex, parental education, physical activity etc.)^30,40^. Similar to other CMD outcomes, sex-driven associations are also present between diet and diabetes, where in the DONALD Study, both dietary sugar intake (*N* = 254) and flavonoids from fruits and vegetables (*N* = 241) were positively associated with homeostasis model assessment insulin sensitivity (HOMA2-%S) among females^41,42^. This supports the potential moderating role of sex in adulthood diabetes development from adolescent dietary intake, prompting more longitudinal studies examining the sex-stratified associations in the future.

Importantly, studying a combination of risk factors can be more predictive of CMD development than individual outcomes due to the advantage of detecting subtle cumulative risks amongst a range of factors. While the use of a composite CMR score or metabolic syndrome risk as study outcomes is becoming increasingly popular in epidemiological research, adolescent studies are limited, and sex-stratified analyses are lacking. Metabolic syndrome refers to a group of risk factors that lead to cardiovascular disease and type 2 diabetes, which are the leading causes of death worldwide^43^. Findings from the ALSPAC cohort (*N* = 1940) show that a higher Mediterranean-style diet score and stronger adherence to the UK dietary guidelines as assessed by the Eatwell Guide score during adolescence was associated with reduced CMR score in early adulthood^44,45^. Both these studies adjusted for child sex, dietary misreporting, socioeconomic status, birth factors, and puberty stage, a crucial confounder in adolescent health research. In the Healthy Lifestyle in Europe by Nutrition in Adolescence (HELENA) Study and the follow-up Better Life by Nutrition During Adulthood (BELINDA) Study (*N* = 164), after adjusting for child sex, study city, alcohol and selected nutrient intakes, a higher lignan (a group of polyphenol) intake was found to be significantly associated with lower risk of adult metabolic syndrome, as defined by the American Heart Association^46^.

The observed sex-driven differences in existing literature may arise due to physiological distinctions such as hormonal regulation (e.g., estrogen, IGF-1) or behaviorally influenced dietary patterns. While these influences may be stronger during pubertal development, many studies do not account for puberty staging or status in their model adjustment. In some studies, different statistical models (e.g., not all studies adjusted for physical activity, socioeconomic status, or baseline health outcomes), residual confounding, or inadequate power in sex-stratified analyses may also account for inconsistencies. Lastly, observed discrepancies between studies often reflect methodological variations, such as exposure definitions (e.g., hypothesis-driven dietary indices, data-driven dietary patterns), dietary assessment tools (e.g., food frequency questionnaires, 24-hour dietary recall), and population characteristics (e.g., geographically driven dietary patterns, different dietary guidelines across countries), rather than true contradictions.

## Potential adolescent diet-disease mechanisms

Given the existing evidence, we hypothesize several mechanisms in which dietary exposures during adolescence can impact the development of CMDs.

### Pubertal development

Temporal changes in the timing of puberty are supported by the DOHaD hypotheses which suggest that poor health and undernutrition lead to delays in full reproductive capability. Current literature supports a divergent secular change in girls as a result of an increased prevalence of obesity; younger age at puberty onset but no change in age at menarche^47,48^. Nutrition, environmental, genetic and psychological factors can interact with pubertal pathways. Additionally, endocrine-disrupting chemicals, found in ultra-processed foods, can affect energy balance and the timing of puberty^49,50^.

**Fig. 1:**
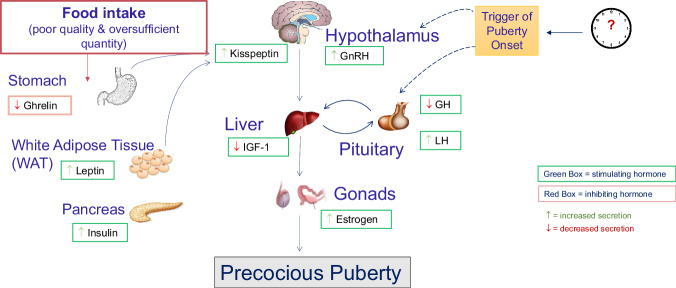
Overnutrition and puberty mechanism of action. Normal puberty onset is controlled by the interaction between stimulating hormones (green box) and inhibiting hormones (red box). The effect that poor and insufficient intake has on these hormones is indicated by the arrows. Green arrows () indicate an increased secretion and red arrows () indicate decreased secretion in states of undernutrition. IGF-1 Insulin-like Growth Factor-1, GH Growth Hormone, GnRH Gonadotropin-releasing Hormone, LH Luteinizing Hormone, IGFBP-1 Insulin-like Growth Factor Binding protein-1.

Nutrition impacts hormonal production, which influences puberty onset timing and the epiphyseal growth plate. Current understanding considers energy sufficiency or insufficiency in signaling the HPG-axis in puberty onset timing^51^. Additionally, a higher intake of animal protein is correlated to earlier puberty growth compared with a higher intake of vegetables associated with later puberty onset^52^. Early puberty onset is associated with adverse health outcomes such as metabolic syndrome and cardiovascular disease^53^. Of particular importance are leptin, ghrelin, Kisspeptin, adenosine monophosphate-activated protein kinase (AMPK) and the mammalian target of rapamycin (mTOR). Leptin levels are directly related to adipose stores and act within the hypothalamus to adjust energy requirements. The gradual increases in GnRH are highly sensitive to body energy reserves that are influenced by leptin. In overnutrition, excess levels of leptin permit the gradual increase of GnRH leading to earlier puberty onset^54^, whereas in cardiometabolic undernutrition (Fig. 1), leptin levels are low and no such permissive action exists. Ghrelin is secreted from the stomach as a result of energy insufficiency and acts as an inhibitory control on puberty onset in girls^55^. Kisspeptin also stimulates, directly and indirectly, the GnRH neurons that drive HPG axis maturation and puberty onset.

**Fig. 2:**
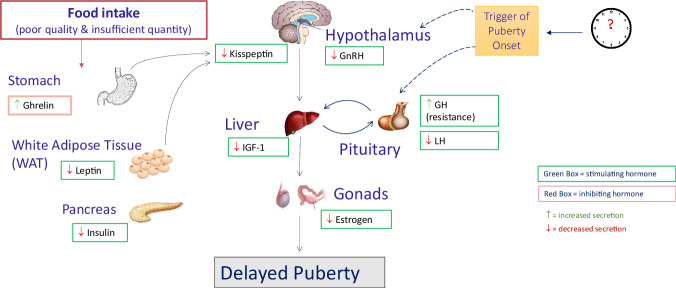
Undernutrition and puberty mechanism of action. Normal puberty onset is controlled by the interaction between stimulating hormones (green box) and inhibiting hormones (red box). The effect that poor and insufficient intake has on these hormones is indicated by the arrows. Green arrows () indicate an increased secretion and red arrows () indicate decreased secretion in states of undernutrition. IGF-1 Insulin-like Growth Factor-1, GH Growth Hormone, GnRH Gonadotropin-releasing Hormone, LH Luteinizing Hormone, IGFBP-1 Insulin-like Growth Factor Binding protein-1. Reprinted with permission from Campisi, Susan; Carducci, Bianca; Söder, Olle; Bhutta, Zulfiqar. The Intricate Relationship between Chronic Undernutrition, Impaired Linear Growth and Delayed Puberty: Is ‘catch-up’ growth possible during adolescence? Innocenti Working Papers no. 2018-12.

Undernutrition also decreases the secretion of hormones that promote epiphyseal growth plate elongation and increases the secretion of hormones that inhibit epiphyseal growth plate elongation (Fig. 2). Additionally, nutritional adequacy during the pubertal growth spurt is paramount. Adequate bone mineralization during puberty requires the essential building blocks of bone. Food intake provides skeletal constituents like calcium, phosphorus, zinc, vitamin D, vitamin A and protein to support bone growth. Zinc deficiency can severely impact zinc-dependent collagenolytic proteases that facilitate changes in matrix proteins. These proteins allow for bone deposits and thus decrease elongation in the hypertrophic zone of the growth plate –the stage which adds the greatest contribution to variability in growth. In addition, vitamin D metabolites regulate the hedgehog pathway both as morphogenetic inducers and repressors^56^. Specific amino acids act on chondrocyte maturation in the proliferation zone by disrupting or inhibiting growth plate elongation when they are in limited supply^57,58^. When an adolescent’s diet lacks the essential building blocks of bone during puberty, optimal bone proliferation and elongation will not be accomplished.

While Figs. 1 and 2 depict the physiological effects of pronounced undernutrition or overnutrition on puberty-related hormonal pathways, these scenarios reflect extreme ends of the nutritional spectrum. The majority of adolescents experience habitual dietary patterns that may include mild nutrient deficiencies or excesses, such as suboptimal intake of iron or protein, which can still influence growth trajectories and pubertal timing over time^8–13^. These subtler, but chronic, dietary imbalances may not immediately manifest as clinical malnutrition but can cumulatively affect hormone regulation, body composition, and long-term cardiometabolic health.

Importantly, weight status during adolescence (e.g. including those with overweight or obesity, or being underweight) may act as a confounder, mediator, or effect modifier in the association between adolescent diet and adult CMD risk. For instance, higher BMI may alter hormone levels (e.g., leptin, insulin) that influence pubertal timing, while also independently affecting CMD risk. Therefore, future research should account for weight status in analytical models to better isolate the role of nutrition and to clarify the complex interplay between diet, pubertal development, and later-life disease.

### Epigenetics

Early life exposures can leave a long-term imprint on disease susceptibility through epigenetics – heritable changes in gene expression independent of DNA sequence^59^. Key mechanisms include DNA methylation, which acts as a dimmer switch for genes, and its interaction with single nucleotide polymorphisms. These variations in the DNA code can influence methylation patterns, further modulated by early life experiences like environment and nutrition. This interplay ultimately shapes gene expression and potentially programs disease risk later in life.

A recent study explored changes in DNA methylation pre- and post- adolescence considering physiological exposures such as BMI, age when growth spurt started and age at first menarche as well as pharmaceutical exposures such as smoking, paracetamol use and non-steroidal drugs^60^. They identified a vast number (>15,000) of DNA methylation sites that changed regardless of sex at birth (hereafter referred to as sex) while over a thousand sites displayed sex-driven methylation changes relevant to cell growth and immune system development. Among physiological factors, pre-adolescent BMI in females and growth spurt timing in both sexes were associated with DNA methylation changes during puberty. Notably, non-steroidal drug use and smoking emerged as the pharmaceutical exposures with the most significant impact on DNA methylation patterns. These findings highlight the dynamic interplay between early life exposures, adolescent experiences (including puberty), and the epigenome, ultimately shaping an individual’s susceptibility to CMDs.

### Microbiome

Recent research suggests the gut microbiome, a complex ecosystem of trillions of microbes residing in our gut, acts as a crucial link between DOHaD, adolescent health, and the development of CMDs later in life^61^. A critical window in early life exists for establishing a healthy microbiome, impacting future health. Birth mode, diet, and antibiotics can influence this microbiome, potentially affecting disease risks (asthma, obesity, etc.). This aligns with the core principles of DOHaD^62^. Lee et al. explored how early life experiences, particularly maternal diet, can significantly influence the developing gut microbiome through microbial metabolites like short-chain fatty acids and vitamins^63^. These metabolites act as messengers, influencing epigenetic modifications in the host’s cells. This process involves changes such as histone acetylation, which can activate or silence genes involved in various functions like immune system regulation and metabolism, potentially affecting the offspring’s health throughout life. For instance, maternal high-fat diets may lead to alterations in the gut microbiome and epigenetic changes, increasing susceptibility to obesity and metabolic disorders later in life.

Diet plays a significant role in shaping the gut microbiome throughout life. The types of food consumed, including various macro- and micro- nutrients, provide substrates and influence the growth and function of different microbial communities^64–66^. This relationship between diet and the microbiome starts in infancy. Studies have shown significant differences in bacterial colonization patterns between breastfed and formula-fed infants^67–70^. These differences continue as infants transition to solid foods^71^, and persist through adolescence and adulthood^72^. While most research has focused on infant diet-microbiota interactions, recent longitudinal studies demonstrate that the gut microbiome remains sensitive to dietary changes throughout adolescence^73^. Notably, the effects of these dietary shifts can persist into adulthood^74^.

Beyond its well-established roles in digestion and immunity, the gut microbiome is emerging as a novel regulator of estrogen bioavailability. Certain gut microbes, particularly those producing β-glucuronidase (e.g., Ruminococcus and Faecalibacterium), can influence estrogen metabolism. This gut-estrogen connection, observed in both human and animal studies, coupled with the impact of dietary fiber on estrogen levels, suggests the potential of the microbiome to influence puberty timing and development. This opens doors for exploring novel DOHaD therapeutic targets^75,76^.

Recent research by Korpela et al. investigated the relationship between pubertal timing and the gut microbiome^77^. They compared fecal microbiota composition in pre-pubescent, pubescent, and adult individuals. Their findings revealed a novel correlation between prepubertal antibiotic use and delayed puberty onset. Additionally, they observed a shift in the gut microbiota towards an adult-like composition as puberty progresses. This shift impacted specific bacterial species, suggesting a potential influence of puberty on gut microbiota composition. Interestingly, the observed gut microbiota changes were more pronounced in girls compared to males. This discrepancy might be due to later male puberty or limitations in the study design. Nevertheless, these findings, particularly the link observed in females, suggest a potential interaction between gut microbiota and sex hormones during puberty. Further research exploring early-life exposures, the adolescent microbiome, and sex-driven interactions can provide deeper insights into how DOHaD influences CMD development.

### Metabolome

A deeper understanding of the adolescent metabolome may provide insight into the intricate interplay between metabolic pathways like oxidative stress, inflammation, and type 2 diabetes development. Oxidative stress is associated with diets high in free sugars that induce postprandial hyperglycemia^29,40,78^. One study hypothesized that fiber intake may affect fat oxidation when discussing the association between high fruit and vegetable fiber intake and adverse effects for weight and body fatness^34^. Greater adherence to the Mediterranean diet has also been hypothesized to be protective against oxidative stress, due to its balanced nutrient quality^36,44^. It is thought that excessive oxidative stress could impair mitochondrial functions within hepatocytes, thereby increasing insulin resistance and promoting adverse health outcomes^40^. In addition, inflammation, through cytokines and oxidative stress, work concurrently in the development of CMD outcomes^26,78^. From the DONALD Study, a high intake of high-glycemic index foods and low whole grains during adolescence was associated with greater IL-6 concentrations in early adulthood^78^. Additionally, this study found that a higher intake of flavonoids from fruits and vegetables^42^, but not dietary sugar intake^41^, during adolescence was associated with a pro-inflammatory score (comprised of CRP, IL-6, IL-18, leptin, chemerin, and adiponectin) in adulthood, an indicator hypothesized to be more predictive of inflammation. Certain nutrients, such as flavonoids, can exert anti-inflammatory and antioxidant effects by NF-κB inhibition, and improving insulin sensitivity^42^.

Females tend to have more dynamic hormone regulation than males, making them more susceptible to dietary influences and lowering their insulin sensitivity^41^. Female sex hormones may also play a role in regulating sodium excretion, allowing for better control of blood pressure when exposed to a high-salt diet^39^. Lastly, growth hormones, such as IGF-1, may be upregulated in adolescence, and in combination with insulin resistance at puberty onset, they may work synergistically in the development of obesity in later life^29^.

## Interventions for health promotion and cardiometabolic disease prevention

Building on the DOHaD framework, in order to redesign, promote and integrate interventions across the life course and the continuum of care of children and adolescents to both promote healthy diets and prevent CMDs, requires an understanding of the multiple ‘layers of the environment’ in which we develop^79–81^. These environmental layers include the intrauterine environment, our familial histories, our household conditions, our communities, and the socio-political and economic structures in which we are embedded^81^. By recognizing this ecological nature, multisectoral interventions, across systems and levels can be designed with an understanding of mechanisms, pathways, and interactions between risk and protective factors, as well as child and adolescent values and social context, to ultimately protect child and adolescent developmental trajectories and human capital^82–85^.

From a food systems perspective, improving food environments – or the collective physical, economic, policy, and sociocultural surroundings, opportunities and conditions that influence people’s food and beverage choices and nutritional status^86^ is a key platform to improving adolescent diets^87,88^. This includes population-level, food supply chain and market-level interventions directed at improving the regulation of both nutritious foods and foods high in free or added sugars, sodium, trans-fats and additives (i.e., marketing and labeling), nutritional quality and food safety of foods (i.e., reformulation and fortification), as well as the desirability and convenience of nutritious foods. In addition, as emphasized by the Food and Agriculture Organization and World Health Organization (WHO), many countries invest considerable efforts in developing dietary guidelines but pay less attention to implementing monitoring and evaluation processes^89^. Collecting nationally representative nutrition data on adolescents^90^, updating guidelines to meet current dietary patterns, as well as ensuring compliance in environments in which adolescents interact are critical to designing national guidelines to guide healthy diets.

Within households, food environments serve as an important sphere of influence and point of intervention, particularly around improving caregiver knowledge, attitudes, and practices in food choice through social behavior change communication^91,92^. The transition into adolescence allows for greater autonomy to purchase and consume food outside the home, especially ultra-processed foods^91,92^ with potential influences of peers and conventional and digital advertisements (e.g., social media and Internet marketing)^91–93^. Therefore, health systems interventions such as the promotion of healthy diets and physical activity through nutrition counseling, mass and social media campaigns, micronutrient and food supplementation, management of severe and moderate acute malnutrition, home fortification and deworming should be part of an essential package of adolescent health and nutrition interventions^83–85^.

Additionally, the school environment as a delivery platform remains the most well-researched, particularly around environmental and behavioral change in the prevention and management of cardiometabolic diseases in this age group^23^. One promising integrative strategy, the Health Promoting School approach (or whole-school approach), was developed by the WHO in the 1980s and focuses on a broad spectrum of policies, program activities and services that contribute to the health, safety and well-being of students, staff and families while assuring a supportive and health-promoting environment that nurtures academic growth and development^94,95^. This includes but is not limited to health services, the school food environment (including school meals), health and physical education, counseling, psychological and social services, health promotion training for staff, and family/community involvement^94,95^. While there is strong global guidance, local adoption and implementation at a scale to yield population-level impacts continue to be hampered by a lack of contextually relevant intervention evidence. As such, widespread knowledge translation beyond the research community may help to reshape structures, guidelines, policies and individual behaviors to better the developmental conditions for future generations.

As noted within the Lancet Adolescent Nutrition Series and Optimising Child and Adolescent Health and Development Series, there continues to be a dearth of evidence, especially in LMICs, particularly on the effectiveness of other platforms (i.e., community and digital) to reach this population (especially those out-of-school), the role of factors such as social norms, media and agency, and the full range effects of multifaceted, multisectoral interventions to ultimately support health, wellbeing and development^83–85,96^.

Together, an equity-focused and whole-government approach that delivers healthy diets and reduces cardiometabolic disease is required to improve the immediate conditions in which adolescents are born, live, learn, work and play^97^. Integrating adolescent health financing and programming across the spectrum of maternal and child health activities can enable more effective planning and operational synergies, especially in low-resource settings^98^.

## Future research, policy and practice

Adolescence is a critical life stage where diet-related behaviors are formed, making interventions guided by DOHaD principles essential to curb cardiometabolic disease. Nutrition is one of the most well-studied factors in the human epigenome. However, research on the dynamic mechanisms behind the long-term health effects of adolescent dietary exposures, and how these might differ by sex, is still in its infancy. While conceptual models such as those presented by Hanson and Gluckman acknowledge adolescence as a key developmental stage, few global research agendas, public health strategies, or intergenerational DOHaD initiatives have translated this understanding into actionable frameworks^3^. Our paper aims to bridge this gap by providing concrete evidence and mechanisms linking adolescent nutrition with both current and future health outcomes. We propose that future DOHaD research and programming must explicitly include adolescence in design, funding priorities, cohort studies, and mechanistic work.

First, high-quality longitudinal studies, especially in LMICs, are needed to investigate how nutritional exposures during adolescent influence the development of cardiometabolic diseases in adulthood. This also includes intergenerational studies to determine the intergenerational effects of adolescent nutrition by examining how parental nutrition during adolescence affects the health outcomes of future generations. Sex-driven effects also need to be investigated to understand whether nutritional exposures during adolescence have differential effects on males and females in terms of disease susceptibility, metabolic health, and developmental trajectories.

Second, nutritional interventions targeted at adolescents are needed to optimize health outcomes across the life course. This will evaluate the efficacy of dietary interventions, supplementation strategies, and lifestyle modifications in improving metabolic health, reducing disease risk, and promote overall well-being. In addition, food environment factors need to be considered to investigate how access to nutritious foods and food insecurity influence dietary patterns, nutritional intake, and health disparities during adolescence and beyond.

Third, while we discuss potential mechanisms, further studies are needed to investigate the multifaceted mechanistic pathways underlying the relationship between adolescent nutrition and health outcomes. This mechanistic exploration should encompass epigenetic modifications, metabolomic profiles, and microbiome composition as interconnected systems that may mediate the long-term effects of adolescent nutritional exposures. Current population-based cohorts with longitudinal follow-up present valuable opportunities for tracking these mechanistic markers, allowing researchers to examine temporal relationships between adolescent dietary patterns and molecular signatures that precede clinical outcomes. Future mechanistic investigations, in both humans and animal models, should prioritize nutrient signaling pathways, metabolic programming mechanisms, and inflammatory cascades, examining how these systems interact during adolescence to establish metabolic risk thresholds that influence cardiometabolic disease risk across the lifespan.

In addition to studying these mechanisms, future research must place greater emphasis on the physiological and endocrine context of adolescence. This developmental window is marked by significant inter-individual variation in hormonal levels (e.g. testosterone, IGF-1, leptin, insulin, and cortisol) which can directly influence growth, metabolism, and inflammation, and may strongly mediate or modify the effects of dietary exposures. These intrinsic hormonal differences, whether genetically or environmentally driven, may mask or amplify the associations between diet and cardiometabolic outcomes. Therefore, measurement and adjustment for these endocrine markers is critical in longitudinal cohort studies to disentangle the independent effects of adolescent nutrition.

Particularly when adolescence is examined as a sensitive period in DOHaD-informed research, future studies should aim to systematically include endocrine confounders or mediators in statistical models, both to clarify causal inference and to unmask potentially modifiable nutritional pathways. This is especially relevant for mechanistic interpretation, where mild or moderate dietary imbalances may otherwise be difficult to detect without accounting for underlying physiological variability.

Lastly, research findings need to be translated into evidence-based public health policies and interventions aimed at improving adolescent nutrition and preventing CMDs in adulthood. Advocacy for policies that promote healthy eating environments, nutritional education, and access to nutritious foods for adolescents will be a critical component of this effort. Collaborative research networks also need to be established to foster collaboration and knowledge exchange among researchers, healthcare professionals, policymakers, and community stakeholders to advance research on adolescent nutrition from a developmental origins’ perspective.

## Conclusion

Our contribution builds on existing DOHaD literature that recognizes adolescence as a biologically significant window of life. We also recommend the explicit, programmatic, and mechanistic integration of adolescence into DOHaD research priorities, global implementation frameworks, and intergenerational health promotion efforts.

Investing in nutrition throughout critical stages of development, including adolescence, is essential for reaping both short-term and long-term benefits^99^. It can lead to significant economic and social advantages, including reduced health care costs, improved educational outcomes, and enhanced productivity^99^. Several evidence-based strategies have been suggested to achieve these goals, but there is a need for investments in large-scale program implementation, with better monitoring and evaluation, including data that is age- and sex-specific. Some of these strategies are food-based, such as dietary diversification and food fortification or behavior-based, achieved through school-based nutrition education interventions, social media, and mobilizing families and communities. Finally, from both CMD prevention and DOHaD preconception lens, there is a need to prevent nutrition deficiencies, by conducting regular nutrition assessments and providing specialized counseling to adolescents. By addressing the gaps in both research and practice in adolescent nutrition, we can develop more effective programs which can contribute to better overall health and well-being of adolescents and help them reach their full potential. Ultimately, closing these gaps can benefit society, leading to improved population health and economies.

## Data Availability

Data sharing is not applicable to this article as no datasets were generated or analyzed during the current study.

## Code availability

Code sharing is not applicable to this article as no code was generated during the study.
